# Machine-Learning-Enabled Tensile Property Prediction of Fused-Filament-Fabrication-Printed Recycled PLA/Wood Composites Fabricated via Solution Casting

**DOI:** 10.3390/polym18151820

**Published:** 2026-07-25

**Authors:** Venkata Durga Sahithi Vaka, Dhanunjay Kumar Ammisetti, Kruthiventi Sai Sarath, Priyaranjan Samal, Ravi Kumar Kottala, Seepana Praveenkumar, Jamal-Eldin F. M. Ibrahim

**Affiliations:** 1 Department of Mechanical Engineering, VNR Vignana Jyothi Institute of Engineering and Technology, Hyderabad 500080, India; sahithi_vvd@vnrvjiet.in; 2 Department of Mechanical Engineering, Lakireddy Bali Reddy College of Engineering, Mylavaram 521230, India; adkphdnitt@gmail.com; 3 Department of Mechanical Engineering, Koneru Lakshmaiah Education Foundation, Vaddeswaram 522502, India; sharath@kluniversity.in (K.S.S.); ps23@iitbbs.ac.in (P.S.); 4 Interdisciplinary Research Center for Construction and Building Materials, King Fahd University of Petroleum & Minerals (KFUPM), Dhahran 31261, Saudi Arabia; jamalfadoul@gmail.com; 5 Department of Nuclear and Renewable Energy Sources, Ural Federal University Named After the First President of Russia B. N. Yeltsin, 620002 Yekaterinburg, Russia

**Keywords:** polymer composites, sustainable composites, fused filament fabrication, mechanical optimization, machine learning, statistical optimization

## Abstract

This study investigated the manufacturing and impact of critical input parameters in fused filament fabrication (FFF) on the ultimate tensile strength (UTS) of tailor-made recycled PLA/wood bio-composite specimens. As technology advances rapidly, several wood-based polymer composites have emerged as promising materials for wood-based interior applications. In the current work, recycled PLA material is combined with wood powders to form composite 3D printing filaments. The solution casting method is used to recycle the PLA and a single-screw extruder is used to fabricate the composite filament. 3D printing parameters play a major role in enhancing the characteristics of the wood-based polymers. This study considers the printing temperature (PT), layer height (LH), and printing speed (PS) as input parameters at five levels. Taguchi Design of Experiments (L25 orthogonal array) was employed to minimize experimental runs, followed by ANOVA analysis to find influencing factors. The results demonstrated that layer height (83.91% contribution) is the most critical parameter, with 0.1 mm identified as the optimal amount for achieving the maximum UTS response, while printing temperature (2.47%) had a moderate effect and printing speed (2.28%) showed negligible influence. In the present work, the tensile properties of the composite filament were predicted using machine learning methodologies, including random forest (RF), support vector regressor (SVR), Gradient Boosting Regression (GBR), Extreme Gradient Boosting (XG Boost), and Adaptive Boosting (Adaboost). The results indicate that support vector regressor (SVR) outperformed all other models in terms of generalization, as it generated the lowest test errors (mean squared error (MSE) = 0.0169, mean absolute error (MAE) = 0.0953, mean squared logarithmic error (MSLE) = 0.0065 and mean absolute percentage error (MAPE) = 0.2246) and the highest predictive power (coefficient of determination (*R*^2^) = 0.8679).

## 1. Introduction

The advent of Industry 5.0, characterized by a fusion of human creativity, sustainable practices, and resilient smart systems, is redefining manufacturing paradigms [1]. Unlike Industry 4.0’s focus on automation and interconnectivity, 5.0 highlights human–machine collaboration, eco-efficiency, and socially responsible production [2]. Within this framework, 3D printing, particularly FFF, has emerged as a cornerstone technology, enabling decentralized, customizable, and waste-minimized manufacturing [3]. In this context, 3D printing plays a critical role by lowering material waste and energy utilization compared to traditional manufacturing methods. Unlike subtractive processes that generate significant waste, 3D printing only uses the required quantity of material needed, making it an eco-friendlier option. The most sustainable materials used in 3D printing are PLA (Polylactic Acid) and PLA/wood composite filaments. PLA, a biodegradable plastic made from renewable resources like cornstarch or sugarcane, not only reduces environmental impacts but also emits fewer toxic fumes during the printing process, contributing to a cleaner manufacturing environment. By combining PLA with finely ground wood fibers, the PLA/wood composite filament combines the advantages of sustainability and esthetics together. This material allows for the creation of products with a natural wood-like appearance, offering an eco-friendly alternative for applications such as furniture, interior decor, and prototypes [4]. However, there are many challenges involved, such as weak interfacial bonding and the anisotropic behavior of the printing. Optimization of process parameters is the most effective approach to maximize output and mitigate associated disadvantages [5]. Wood composite polymers have gained significant interest and importance in research, with many researchers actively contributing to their development due to their wide range of applications, particularly in wood-based interiors. With the advent of 3D printing, which enables the fabrication of intricate and esthetically complicated architectures with relatively low effort, PLA/wood composite filaments have attracted considerable attention for advanced manufacturing applications. The fabricated PLA/wood composite filaments are suitable for selected home furniture and interior applications as they exhibit a wood-like appearance and retain a natural wood aroma due to the presence of wood filler.

Wood-fiber reinforced PLA composite materials exhibited superior mechanical properties compared with virgin PLA resins according to Huda et al. The flexural modulus of a 30 wt% fiber composite material was found to be equivalent to that of traditionally used wood-fiber-reinforced polypropylene (PP) composite materials and was approximately equal to 8.9 GPa, whereas PP composite materials have an average flexural modulus value of about 3.4 GPa. In addition to exhibiting a high flexural modulus, the addition of wood fibers greatly improved the storage modulus (stiffness) of the PLA matrix [6]. According to Ayrilmis et al., the thickness of the printed layers increased the amount of water absorbed into the printed samples; however, the thickness of the printed layers decreased the thickness swell of the printed samples [7]. Due to the increased layer thickness, large interlayer voids formed, causing greater porosity throughout the cross-sectional area of the printed samples. As a result of this increased porosity, the mechanical properties of the printed samples were adversely affected and the strength was reduced. The mechanical properties of printed samples produced using FDM were studied by Kechagias et al. They studied how the LT, RA, and PS influenced the properties of printed parts. Using an ANOVA-based linear regression model to predict the mechanical properties of printed samples, they determined that the raster deposition angle had the greatest effect on the mechanical properties of printed samples when compared to the other factors evaluated in their study under the same experimental conditions [8]. Using genetic algorithms (GA) and grey wolf optimization (GWO), Fountas et al. investigated the effect of varying control parameters on the UTS of printed samples. Through their research, they demonstrated that GA and GWO can improve the mechanical properties of printed samples [9]. As reported by Bahar et al., the inclusion of wood particles in a PLA matrix resulted in a slight decrease (about 1%) in Young’s modulus. Bahar et al. also stated that as the percentage of infill in a sample was decreased from 40% to 10%, the thermal conductivity of the sample decreased by 37%; however, the thermal diffusivity and thermal effectivity of the sample increased by 21% and 43%, respectively [10]. According to Sultana et al., the mechanical properties of printed components are dependent upon several input parameters that are involved in the 3D printing process. The results of their investigation revealed that LH is the sole parameter with a significant impact on elastic modulus, tensile strength, and maximum load, contributing 63.42%, 69.43%, and 69.43%, respectively [11]. Many other researchers also studied the use of statistical models to optimize the process parameters [12,13,14,15]. Bharath et al. applied analysis of variance (ANOVA) and always better control (ABC) analysis to optimize the process parameters of PLA/wood composites made with 80% PLA and 20% wood. From their results, it was concluded that ABC analysis significantly reduced the prediction errors compared to ANOVA [16]. Mishra et al. studied the effect of flexural properties of the bio-compatible PLA/wood composites fabricated using 3D printing [17]. They employed two types of neural network (NN) models to predict flexural strength. The findings indicated that both RA and PS had a notable effect on flexural modulus and load, with the 90° raster orientation producing the highest modulus value of 3121 MPa. Kartal implemented a machine learning (ML) methodology to enhance the properties of Walnut Shell PLA composite materials that were produced using FFF manufacturing. The mechanical properties of the UTS and elastic modulus were characterized by evaluating them based on four factors: LT, PT, deposition angle (DA), and PS, using the Taguchi L18 approach. This was done to ascertain the extent to which each factor contributed to the characterization. Ultimately, the DA has the most significant impact on the properties of the composite materials, as evidenced by the results of an experiment conducted in accordance with ASTM D638 [18]. The RF regression predicts outcomes with high precision and demonstrates that the method is an effective and eco-friendly approach to optimize the 3D printing of composite materials [19]. Anerao et al. conducted a comparison of ML algorithms for the prediction of mechanical properties of wood/PLA bio-composites using FDM. The objective of this investigation is to utilize experimental data to predict properties using ML algorithms, XGBoost and AdaBoost with python 3. In the prediction of the properties of wood/PLA bio-composite, linear regression (LR), support vector machine (SVM), XG Boost, and AdaBoost were transitional models. XG Boost demonstrated the maximum accuracy among the models, suggesting that it is an exceptionally potent predictor of tensile, flexural, and impact strengths [20]. From the literature, it is identified that the 3D printing input parameters have lot of influence on the output such as mechanical and thermal properties of the specimens fabricated with eco-friendly PLA/wood composite filament. Although the mechanical properties of 3D-printed components are influenced by several printing parameters, such as printing path strategy, raster/road angle, contour/perimeter shells, infill pattern, and infill density (ID), the present study is limited to investigating the effects of printing temperature, layer height, and printing speed. All other printing parameters were kept constant throughout the experiments to isolate the influence of the selected variables. Many researchers applied statistical models to optimize/predict the response variables with respect to input factors. Some researchers applied ML techniques to fit the models, but comparatively, this study is very limited.

The unique aspect of this paper can be attributed to the creation of an environmentally friendly filament from recycled PLA composite, consisting of 30 wt% of teak wood flour produced by solution casting-assisted single-screw extrusion technology, suitable for fused filament fabrication. Unlike earlier studies, which primarily focused on optimizing 3D printing parameters using a limited number of predictive models, the present research integrates the fabrication of recycled PLA/wood composite filaments from laboratory waste through solution casting and filament extrusion with an L25 Taguchi orthogonal array-based Design of Experiments. The study further employs ANOVA to identify the significance of process parameters and develops multiple ML models, including random forest, support vector regression, Gradient Boosting Regression, XG Boost, and AdaBoost, to optimize and accurately predict the ultimate tensile strength of recycled PLA/wood composites. Furthermore, microstructural characterization is performed to establish the relationship between processing conditions, internal morphology, interfacial bonding, and mechanical performance. Overall, the results obtained can be considered as a framework to develop environmentally friendly PLA/wood-polymer composite materials with increased mechanical properties, having great application potential in furniture and interior design due to their lightweight and customizable nature.

## 2. Materials and Methods

### 2.1. Fabrication of Recycled PLA/Wood Composite Filament

In the present work, the fabrication of recycled PLA/wood composite filament composite filaments are developed using PLA and wood powders to enhance the biodegradability of the material. The incorporation of wood contributes to the creation of more sustainable and eco-friendly products, offering benefits such as increased strength, stiffness, density, and improved adhesion to the thermoplastic matrix.

Damaged PLA components collected from the 3D printing laboratory were utilized for recycling. The discarded parts were first mechanically shredded into small pieces and subsequently dissolved in chloroform to obtain a homogeneous PLA solution. The resulting solution was cast onto a flat plate and allowed to evaporate under ambient conditions (open sun drying) to facilitate solvent removal and film formation. Chloroform was chosen as the solvent due to its high solubility for PLA which allows the uniform dissolution of the recycled PLA as well as homogenous dispersion of the teak wood particles while carrying out the solution casting technique. Compared with conventional mechanical recycling, the solution-casting approach provides improved filler dispersion and composite homogeneity. The use of chloroform was conducted within the fume hood while wearing the appropriate Personal Protective Equipment. The waste solvent was collected separately and discarded using the institute’s authorized disposal process.

**Figure 1:**
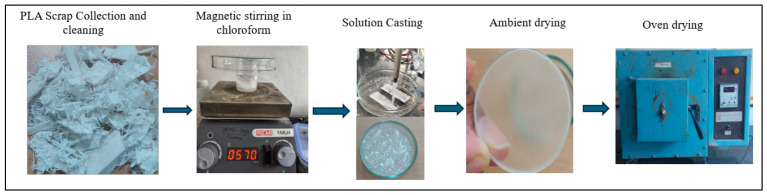
PLA scrap recycling using solution casting.

**Figure 2:**
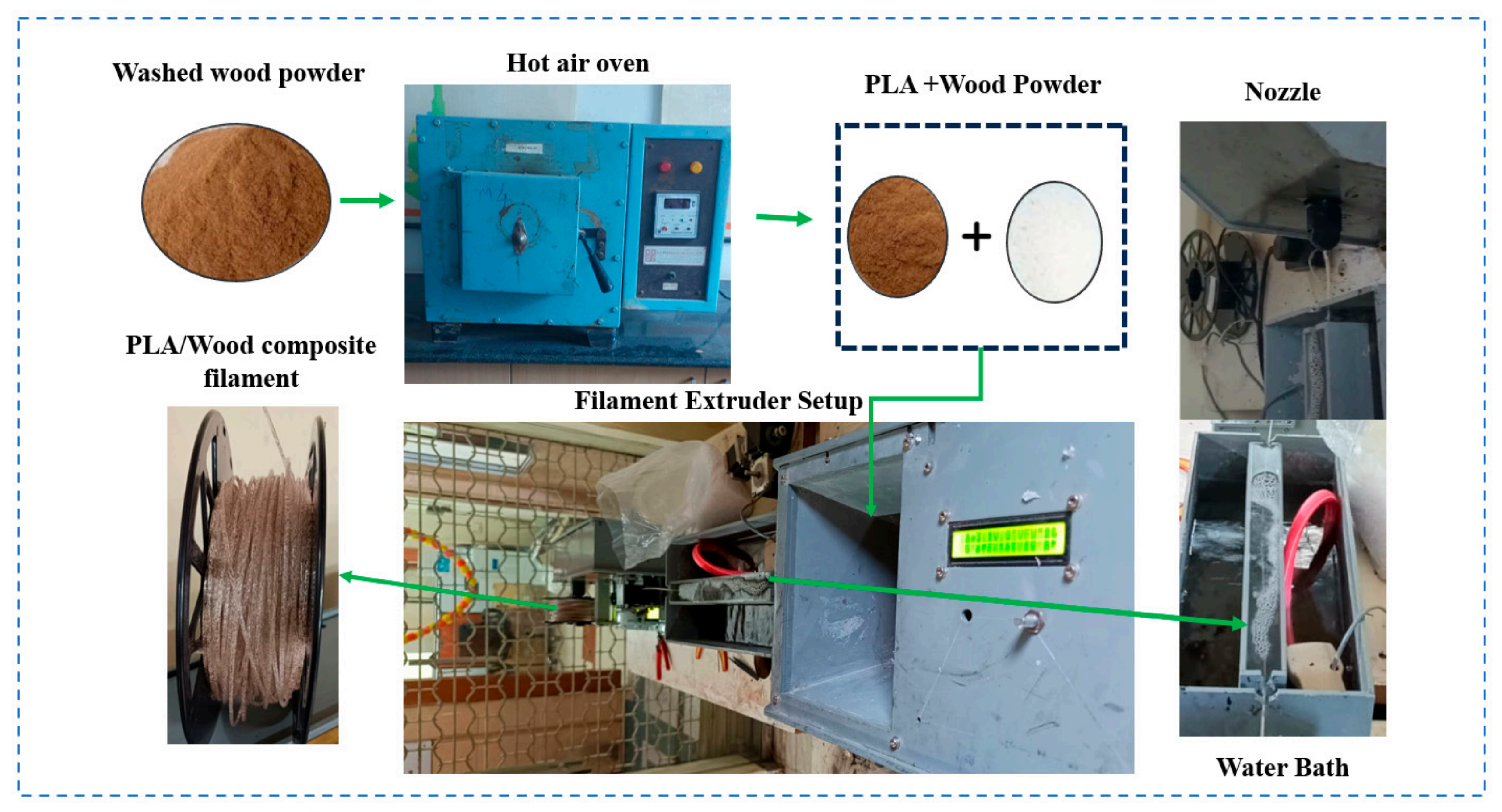
Filament fabrication setup.

After complete drying, a thick PLA sheet was dried by oven heating, then cut into small granules. To eliminate residual chloroform, the granules were further dried in a hot air oven at 40 °C for 4 h prior to subsequent processing as shown in Figure 1. Teak wood powders were obtained from the engineering workshop at VNR VJIET, Hyderabad. To fabricate the recycled PLA/wood composite filament, a composition of 70% recycled PLA and 30% wood powder (WP) was used. Teak wood powder with particle sizes ranging from 50 to 100 μm was selected and measured using a vibratory shaker. The powder was thoroughly washed to remove any impurities and subsequently dried in a hot air oven at 80 °C for 8 h to eliminate moisture. This drying step is crucial to prevent any moisture-induced degradation of the polymer during extrusion, which could negatively impact the mechanical properties of the composite. Then recycled PLA + wood mixture was supplied to a single-screw filament extruder shown in Figure 2. Initially, the extruder temperature was elevated to 200 °C for 30 min to confirm that the internal screw was dry. Then, it was allowed to cool for 20 min and maintained at 170 °C for 10 min before starting of extrusion. The extruded filament was passed through a water bath to promote effective cooling and maintain dimensional stability as shown in Figure 2. Roller speeds were manually adjusted to obtain a steady filament diameter. The final filament produced measured 1.7 mm in diameter with an accuracy of ± 0.05 mm. This filament was then used for 3D printing of test specimens under different combinations of input parameters.

The morphology and surface characteristics of the fabricated composite filaments were examined using Scanning Electron Microscopy (SEM). SEM analysis was carried out using a JEOL JCM-6000 compact benchtop scanning electron microscope (JEOL Ltd., Tokyo, Japan). The observations were performed in secondary electron mode under high-vacuum conditions at an accelerating voltage of 5.0 kV. SEM micrographs were acquired at magnifications ranging from 50× to 500× to evaluate filament quality, porosity, filler dispersion, and the presence of defects generated during the extrusion process. The obtained images were further analyzed to assess the distribution of reinforcement particles and the overall morphological integrity of the fabricated filaments.

### 2.2. Design of Experiments

The mechanical properties of 3D-printed filaments are significantly affected by the selection and optimization of process parameters. In this study, three key input factors were considered, LH, PT, PS, and all were evaluated at five levels to analyze their impact on the final printed specimens. The parameter levels were systematically selected with reference to values and ranges reported in the literature, to ensure relevance and comparability with the previous studies. Input parameters and their levels are shown in Table 1. The layer height levels selected for the study were 0.1 mm, 0.15 mm, 0.2 mm, 0.25 mm, and 0.3 mm. The second parameter, PT, was varied across five levels: 190 °C, 200 °C, 210 °C, 220 °C, and 230 °C. The third factor, printing speed, was evaluated at 30 mm/s, 35 mm/s, 40 mm/s, 45 mm/s, and 50 mm/s. To systematically study the influence of these three parameters at five levels each, a Taguchi L25 orthogonal array design was employed as shown in Table 2. This design methodology effectively reduces the experimental workload from a full factorial design of 125 runs (5^3^ combinations) to just 25 carefully selected combinations. The L25 design ensures that the main effects of each parameter are captured with statistical balance and reliability. Each experimental condition was tested using three specimens, and the reported UTS values were the mean of the three measurements. This approach provides a cost-effective and efficient means of identifying optimal printing conditions while minimizing experimental time and material consumption.

### 2.3. 3D Printing and Mechanical Properties Evaluation

**Figure 3:**
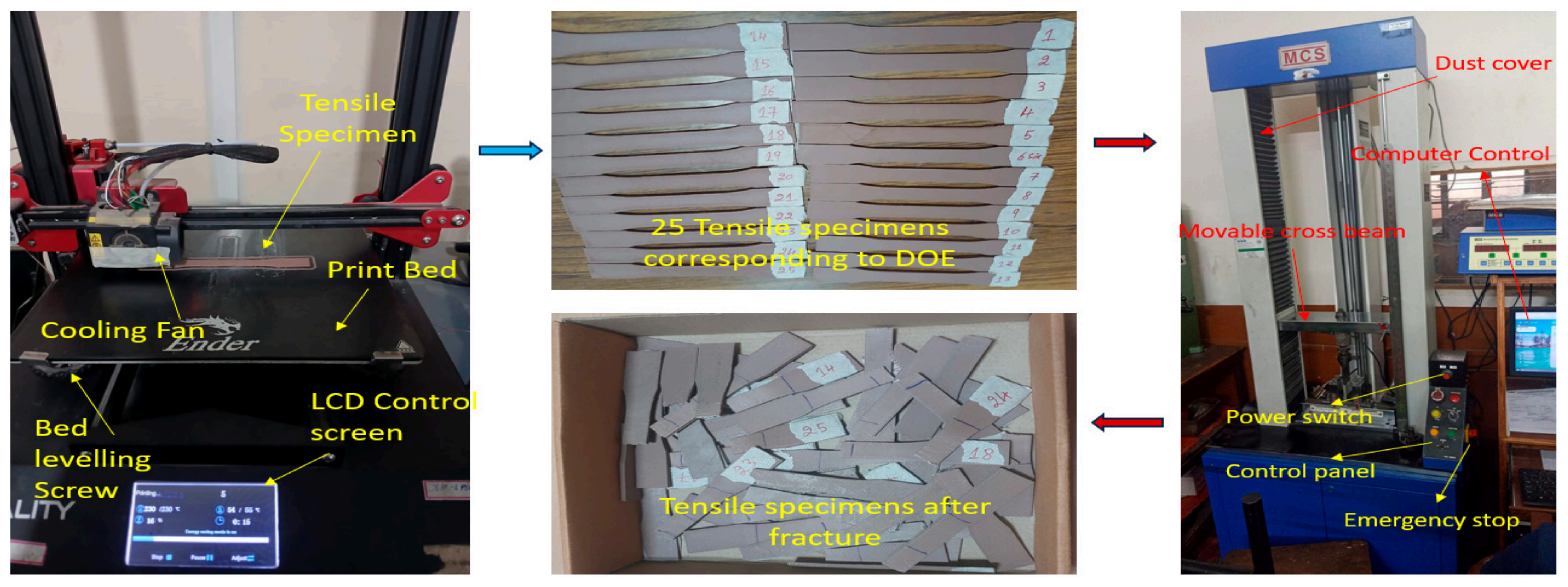
Fabrication and mechanical testing.

After the experimental design, 25 tensile test specimens were fabricated using the recycled PLA/wood composite filament, using ASTM D790 standard [21]. The 3D CAD model of the specimen was created using CATIA V5 software and specimens were printed using a Creality CR-10S Pro V2 3D printer, Shenzhen, China shown in Figure 3. This 3D printer offers a build volume of 300 × 300 × 400 mm and a positional accuracy of ±0.1 mm. This printer supports a slicing thickness range of 0.1 to 0.4 mm, and its maximum PS is 180 mm/s. To achieve a precise and uniform first layer, manual bed leveling was done using the bed leveling screws. Ulti Maker Cura, version 5.10 slicing software was used to create G-codes by inputting the model geometry and parameters such as LH, PS, and PT. This G-code was then transferred to the 3D printer to produce the test specimens. The specimens were fabricated using a Linear infill pattern with an infill density of 75%. The deposition pattern and infill percentage were kept constant throughout all experiments to isolate the effects of the selected process parameters (PT, LH, and PS). These parameters were not included as optimization variables in the present study. After completion of printing, the tensile strength of the fabricated specimens was evaluated using an MCS computerized universal testing machine (UTM) under controlled loading conditions. Figure 3 provides a visual overview of the 3D printing process and mechanical testing setup and the printed specimens.

### 2.4. Statistical Optimization

The methodology of statistically based Taguchi Optimization is a technique that seeks to optimize the performance of an entire process with a minimum amount of experimentation [19,20]. Taguchi Optimization employs orthogonal arrays to test multiple parameters in as few experiments as possible to optimize the target response (UTS). Taguchi Optimization utilizes the Signal-to-Noise (S/N) Ratio to determine which parameter setting has the least sensitivity to variation from trial to trial. Since the goal of this investigation is to maximize the value of UTS, the “Larger-the-Better” S/N Ratio Criterion is utilized. The Larger-the-Better S/N Ratio Criterion applies greater penalty to lower response values. Larger response values equate to larger S/N Ratios, thus providing both high performance and consistency. Equations (1)–(3) represent the S/N Ratios for the three scenarios of the problem. In these equations *p* is the number of trials or experiments and $y_{j}$ is the response for the *j*th observation, $\bar{y}$ represents the mean response and $\bar{s}$ represents the standard deviation of responses.

(1) $$\frac{S}{N}=-10{log}_{10}(\frac{1}{p}\sum_{j=1}^{p}\frac{1}{{y_{j}}^{2}})$$

(2) $$\frac{S}{N}=-10{log}_{10}(\frac{1}{p}\sum_{j=1}^{p}{y_{j}}^{2})$$

(3) $$\frac{S}{N}=10{log}_{10}(\frac{{\bar{y}}^{2}}{{\bar{s}}^{2}})$$

Taguchi Optimization is often used in conjunction with ANOVA (analysis of variance) to provide a deeper analysis of how all the factors interact and affect the output. The primary function of ANOVA is to determine which of the input factors has the greatest effect on the output by quantifying the percent contribution of each factor to the total variance. In addition to determining the percent contribution of each factor, ANOVA will calculate the F-value and *p*-value for each factor. These values are used to determine if there is a statistically significant difference in the mean of the responses due to that specific factor. By combining these two methods of analysis, it is possible to identify and prioritize those factors that are having the greatest impact on the output of the process or product. With this information, it is possible to develop an effective plan for improving the quality of a process or product and for implementing design changes. Therefore, the combination of using Taguchi methods along with ANOVA can be used to improve the reliability and consistency of processes and products.

## 3. Machine Learning (ML) Modelling

In the current research, several supervised ML models have been applied in order to predict the tensile behavior of PLA/wood composite materials. The most relevant input parameters have been selected from the literature based on their potential influence on the mechanical properties of the composites and these include: LH, PT, and PS. Normalization was performed by employing the min-max normalization method with respect to all inputs so that each feature is scaled uniformly and therefore enables the models to converge and train effectively. The Python 3.13.14 library has been used for the implementation of models such as RF, SVR, GBR, XG Boost, and Adaboost. The accuracy of the developed models has been assessed using the R-squared (R^2^), mean squared error (MSE), mean absolute error (MAE), mean squared logarithmic error (MSLE) and mean absolute percentage error (MAPE) measures which are objective measures of how efficiently the model can generalize to new and unseen samples and provide an objective measure of the accuracy of the predictions made by the models. This section includes the data collection phase; the preprocessing of the collected data; building the models and fine-tuning the hyperparameters of the ML models.

### 3.1. Data Collection

The accuracy of the results generated by the ML model is highly dependent on the quality of the dataset that was utilized to train it. The current dataset used to train the ML model contained a significant quantity of high-quality experimental data (input–output), resulting in the development of an accurate and reliable ML model. This dataset consisted of 25 data points based upon the experimental data collected during this research.

### 3.2. Selection of Features

Feature selection is an approach that can be used in modeling to reduce the number of input variables by retaining only the relevant information and eliminating the superfluous noise found in the data. Multiple 3D printing characteristics are used as predictor inputs in regression models developed for evaluation purposes. For this research, only LH, PT, and PS have been selected as the input parameters (features) from the experimental data. The input parameters examined in this study are those that have been identified through experimental analysis and will influence the output response (UTS). As there was no noise in the data, all input parameters were selected as features without using any feature selection model.

### 3.3. Preprocessing of the Data

Data Preparation is an essential and critical step in the generation of ML Models. A full evaluation of the dataset (containing 25 data points) was conducted to eliminate potential errors caused by the limited sample size of the dataset. Once the data cleansing had been completed, the dataset was split using Python into training data and testing data. Randomization of the dataset prior to splitting allowed for the possibility of eliminating any bias introduced during the data collection process on either the training or test sets. The training dataset was used to train the ML Model, while the testing dataset was used to estimate how well the model performed when presented with new data. The data was split so that the training data would be 80% of the total dataset (20 data points) and the testing data would be 20% (5 data points). All ML Methods used for the UTS prediction method used a training dataset of 20 data points and a testing dataset of 5 data points. Once the model was trained, the test data was then used to predict the outcome for unknown input values.

### 3.4. Normalization

Feature Scaling is a procedure implemented to convert all the independent variables (features) into a similar scale so as to improve the performance of the ML model. Data normalization and data standardization are the two most common techniques of Feature Scaling. The following formula was utilized to ensure that the values were normalized from 0 to 1 in the process of normalizing the dataset. The normalization equation can be expressed as Equation (4).

(4) $$Y_{N}=\frac{Y_{i}-Y_{min}}{Y_{max}-Y_{min}}$$

where $Y_{N}$, $Y_{i}$, $Y_{max}$ and $Y_{min}$ are normalized value, ith data value, maximum data value, and minimum data value, respectively.

### 3.5. Various Models Development

#### 3.5.1. Random Forest

The random forest (RF) uses a set of decision tree algorithms to combine the results of individual data points into predictions or classifications. By using multiple decision trees, RF avoids the shortcomings associated with classification and regression tree models. Instead of relying on a single classification or regression tree to make predictions, RF aggregates the results from *m* different classification or regression trees which were generated by creating m bootstrap samples of size *n* from the same training database. At each iteration of generating the m trees, a random subset of *n* predictors is chosen to be used in the construction of the tree as opposed to all predictor variables. Using a specified number of iterations, *m* trees are produced and then estimates from all m trees are combined to produce the output information. Bagging is also employed by RF to create diverse trees and thus to reduce the total variance of the model. The equation below represents the output model of RF in the form of Equation (5).

(5) $$Y_{RF}^{N}\left(x\right)=\frac{1}{N}\;\sum_{n=1}^{N}{DT}_{j}(x)$$

where x is the input vector, *N* is the total number of trees, and ${DT}_{j}$ is the individual tree.

#### 3.5.2. Support Vector Regression

The support vector regression (SVR) is a type of supervised learning algorithm that identifies the minimum margin(s) that minimize the discrepancy between predicted and actual values. The SVM identifies the minimum margin, using the boundary line points called support vectors, to achieve margin optimization. SVM’s robustness to outliers and bias in data allows SVM to converge to an optimal solution faster than most other types of ML methods. Additionally, SVM’s hyperparameter customization options provide a wide range of adaptable solutions to solve many different problems. SVM uses data to create a function that best describes the data while ensuring that the function does not exceed a predetermined margin of error, or epsilon boundary. The SVR ensures that predictions are closely aligned to the actual values and that there can be some acceptable errors. The primary SVR equation, which represents the relationship between simplification and precision, is shown below in Equation (6).

(6) $$f\left(z\right)=\beta^{T}X+b$$

where b is the bias, X is the input, and β is the weight vector.

#### 3.5.3. Gradient Boost Regression

The decision tree model tends to over-fit. Over-fitting is typically prevented by using decision trees where many decision trees are employed as a single model. As a result, these combined models have greater predictive power than decision trees alone. Gradient-boost (GB) algorithm uses an ensemble of multiple decision trees which collectively utilize the boosting method to efficiently combine their individual predictions. Boosting is a systematic way of combining many poor learners into a strong learner. The GB model employs decision trees as poor learners with each tree within the model attempting to correct for the mistakes of the previous tree. Using poor learners like decision trees with the boosting method allows for very high levels of both efficiency and accuracy. Additionally, the GB model improves performance by incrementally adding additional trees one at a time, with each successive tree focusing on areas that were poorly predicted by the previous tree. To increase the methodology’s decision tree efficacy, statistical techniques are applied. The main rationale behind this strategy was to combine several weaker models to create a more robust model. By minimizing the residuals, the GB technique iteratively builds more decision trees. To increase forecast accuracy, an iterative process gradually incorporates a unique tree that minimizes the loss function. Let $\left(x_{1},y_{1}\right),\left(x_{2},y_{2}\right),\ldots \ldots \left(x_{i},y_{i}\right)\ldots \ldots \ldots \ldots (x_{n},y_{n})$ be the data points. The loss function is represented by Equation (7).

(7) $$L\left(y,f\left(x\right)\right)=\sum_{i=1}^{n}{\left(y_{i}-f(x_{i})\right)}^{2}$$

#### 3.5.4. Extreme Gradient (XG) Boost Regression

XGBoost is an ensemble learning algorithm that uses gradient boosting to combine a fixed number of weak learners to increase the accuracy of predictions. In a regression task, the algorithm fits models (trees) stage-wise. It can start with an arbitrary prediction, usually the mean of the target variable, and build trees sequentially. Specifically, iteration one (the first tree) will predict the original observations (provided in the dataset), and the next iteration will train a new tree on the residuals/errors from the first tree, which is the difference between the original observations and the predicted values from the first tree. Therefore, the iterations can repeat many times, and, at each subsequent iteration, the new tree will incorporate the first derivative (the gradient value) from the loss function, whether it is the mean squared error (MSE) or other errors. To improve the predictive performance and overcome over-fitting, XGBoost introduced their own regularization and second-order optimization method to the original form of gradient boosting. The crafting of trees in the original form was guided by gradients of the loss function (the derivative function) while in XGBoost, the algorithm used first (the loss function’s) and second derivatives to craft new trees. The XGBoost algorithm also uses a regularization term (as depicted in Equation (8)) to penalize trees with more complexity. The predicted value is the sum of the outcomes of all trees (as represented in Equation (9)).

(8) $$L\left(\phi \right)=\sum_{i=1}^{n}\left[l\left(y_{i},{\hat{y}}^{\left(t\right)}\right)\right]+\sum_{k=1}^{t}\varphi \left(f_{k}\right)$$

(9) $${\hat{y}}_{i}=\sum_{m=1}^{N}f_{m}(x_{i})$$

Here, ${\hat{y}}_{i}$ is the predicted value at instance i, N is the total number of trees and $f_{m}$ is the decision tree function learned at the mth iteration.

#### 3.5.5. Adaptive Boosting Regression

AdaBoost is a robust ensemble learning technique that merges several weak learners, often decision stumps (one-level decision trees), to create a strong regressor. AdaBoost executes as follows: it trains a weak learner, then trains a second weak learner; however, the second learner focuses more on the training instances that it found to be difficult to predict correctly. This process continues until a stopping criterion is met and the final regressor is a weighted sum of the outputs of every learner. The weights are determined by the performance of each learner when examining the weighted error of the training samples. The algorithm focuses on training models, and every new model increases the weight for instances misclassified in earlier models; hence, AdaBoost focuses on misclassified instances. AdaBoost applies weight to training examples. In later iterations, the algorithm examines how the weak learner performed, the weighted errors and performance, and is based on weighted errors, classifying examples based on the weighted average of the base learners’ outputs. The output function is shown in Equations (10) and (11). AdaBoost, while sensitive to noise, is highly accurate and generalizes effectively in most cases when the regularization of base learners is effective.

(10) $$\hat{y}(x)=\sum_{m=1}^{N}\alpha_{m}f_{m}(x)$$

(11) $$\alpha_{m}=\ln\left(\frac{1}{\beta_{m}}\right)$$

Here, N is the total number of trees, $f_{m}$ is the decision tree function learned at the mth iteration, $\hat{y}(x)$ is the predictive function, and $\beta_{m}$ is the performance score.

### 3.6. Hyperparameter Optimization and Cross-Validation

Hyperparameters denote the variables expressly defined to regulate the learning process before implementing an ML algorithm on a specific dataset. These are utilized to define the learning capacity and complexity of the model. In this study, the hyperparameters for the tree-based models are chosen based on the existing literature. Following the selection of parameters, hyperparameter tuning, i.e., an optimization process is essential for enhancing the predictive accuracy of ML models, including RF, SVM, GBR, XGBoost, and AdaBoost. To account for the relatively small size of the experimental dataset, in order to increase both the robustness and ability to generalize from the developed predictive models, a 5-fold cross-validation strategy was employed during the training of each model. The hyperparameters for each predictive model were determined through an exhaustive search (grid search) of possible combinations of model parameters. By evaluating all potential parameter combinations prior to model selection, this process removed some degree of variability due to reliance on one particular train–test partition, thereby providing a better estimate of the predictive capability of the selected models.

### 3.7. Justification for Machine Learning Model Selection

Although the experimental dataset consists of 25 observations generated using the Taguchi L25 orthogonal array, such sample sizes are common in materials engineering studies based on statistically designed experiments, where each observation is indicative of a specific combination of processing parameters. This study’s objective was not to generate a high-volume ML model. Instead, it sought to evaluate the ability of state-of-the-art ensemble and kernel-based regression methods to predictively represent the relationship between processing parameter combinations and material property responses within the scope of the provided experimental data. As such, RF, SVR, GBR, XG Boost, and AdaBoost were chosen due to their documented success at capturing complex non-linear interactions between processing parameters and mechanical properties. By providing a comparative analysis among these models, the most suitable method for representing the given experimental data may be identified. Additionally, employing both 5-fold cross-validation as well as grid search-based hyperparameter optimization during the construction of the models will provide improved model robustness and will also minimize the likelihood of over-fitting which exists when training a model on a relatively small dataset. The best hyperparameters for the ML models are shown in Table 3. The following section, Section 4, investigates how certain parameters of an FDM affect its UTS as well as the performance of each ML model.

## 4. Results and Discussion

### 4.1. Effect of Various FDM Parameters on UTS

**Figure 4:**
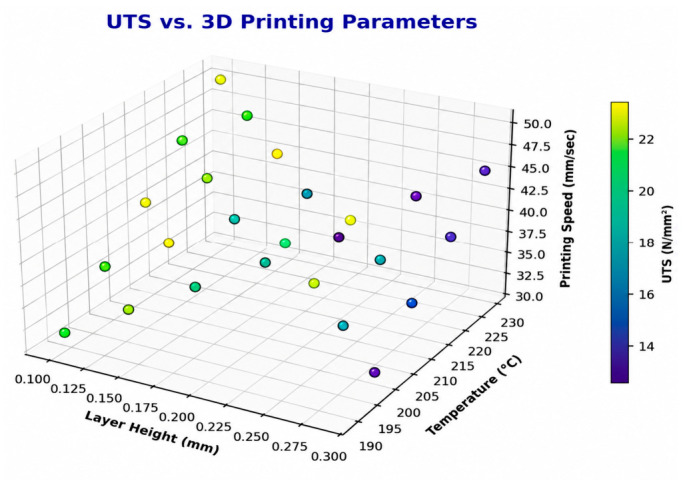
Effect of 3D printing parameters on UTS.

**Figure 5:**
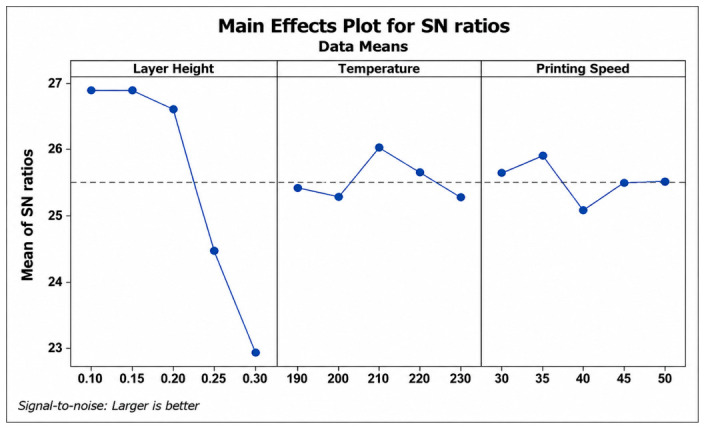
Main effects plots. The blue dots represent the mean S/N ratio at each factor level, the blue lines connect these mean values to illustrate the trend of each process parameter, and the horizontal dashed line indicates the overall mean S/N ratio across all experimental runs.

Ultimate tensile strength is chosen as the key performance metric to evaluate the mechanical properties of the printed samples. To determine the significance and influence of each parameter on the response, the Taguchi approach is supplemented with analysis of variance (ANOVA). Additionally, ML models are applied to predict UTS based on 3D printing parameters (LH, PT, and PS) to identify complex non-linear relationships between inputs and output (mechanical performance). This enables optimization for improved material properties without exhaustive physical testing. Figure 4 shows the 3D scatters plot of UTS values corresponding to L25 experiments. From the response table and main effects plot shown in Table 4 and Figure 5, it was identified that LH is the most influencing parameter with delta value 3.97 and rank 1. In LH, the highest value of the S/N ratio 26.91 corresponds to level 1, which means level 1 (LH = 0.1 mm) corresponds to the optimum LH which leads to highest tensile strength. The lowest value of the S/N ratio 22.94 corresponds to level 5 (LH = 0.3 mm), which leads to lowest UTS value. A lower layer height of 0.1 mm has been found to enhance the UTS of the printed samples. This improvement is mainly due to the finer layer deposition, which promotes stronger bonding between layers. Thinner layers provide a greater contact surface between adjacent layers, leading to better adhesion and improved overall structural integrity. PS is the second influencing factor with delta value = 0.81. The highest value of S/N is 25.95, corresponding to level 2 (PS = 35 mm/s) of PS. The UTS of a 3D-printed part is highest at an optimum PS because it strikes the right balance between layer bonding quality and printing efficiency. From the response table for S/N ratios, PT is the 3rd influencing factor with a delta value of 0.73. Here, the highest S/N value 26.06 corresponds to level 3 (210 °C) of PT and the lowest S/N value 25.32 corresponds to level 5 (230 °C). High printing temperatures can degrade the material and cause weak, inaccurate layer deposition. Low temperatures lead to poor interlayer bonding due to insufficient melting of the filament. Both extremes reduce UTS, making 210 °C the optimal temperature for strong parts. This trend is consistent with previous studies, which reported that lower layer heights improve interlayer bonding and reduce internal voids, thereby enhancing the tensile properties of PLA- and wood-PLA-based composites. Similarly, PT has been reported to play a crucial role in improving layer adhesion and mechanical performance, while excessive layer heights adversely affect tensile strength [22,23,24,25,26].

The ANOVA table (shown in Table 5) evaluates the effects of LH, PT, and PS on the UTS of 3D-printed components. The F-value reflects how much a factor influences the result compared to random variation and *p*-value measures the probability that observed effects happened by chance. Higher F-value and lower *p*-value means the factor is more significant. From the ANOVA table, the layer height shows the highest F-value of 22.24 and a *p*-value of 0.0, indicating that it is a highly influential factor on UTS. The high F-value signifies a strong effect relative to experimental noise, while the zero *p*-value confirms the statistical significance of this influence. The 83.91% influence of layer height on the UTS shows that it is the most important factor affecting the response. The percentage influences corresponding to PT and PS are 2.47 and 2.28, respectively, suggesting the lower significance of the influence of these factors on the UTS value. The value S = 1.714 represents the average prediction error. It indicates that predicted UTS values typically deviate from actual values by about 1.714, which is reasonably low given the overall UTS range. The coefficient of determination R-Sq = 89.0%. This value explains that 89% of the variation in the response is explained by the regression model. This reflects a strong model performance, meaning the combined effects of LH, PT, and PS explain most of the variation in UTS.

**Figure 6:**
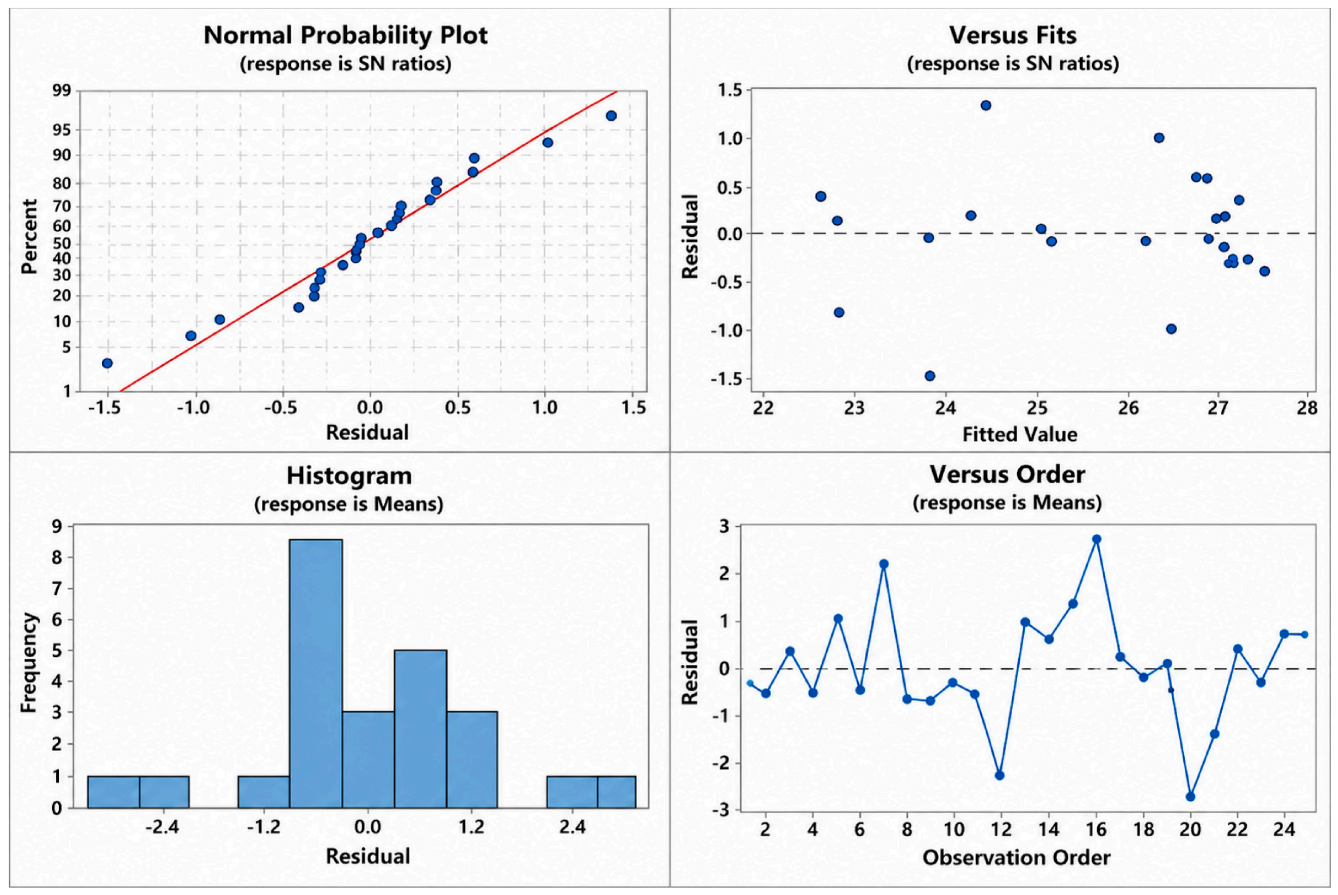
Residual plots for UTS.

The normal probability plot is shown in Figure 6. The fact that every point in the plot is close to the straight line indicates that the residuals and errors follow a normal distribution. There are a few outliers that fall within the permissible range. The residuals vs. fits plot is shown in Figure 6. Every point in this plot is dispersed around 0 with no discernible pattern. The majority of the residuals are at zero with fewer deviations, and the histogram is nearly bell-shaped but somewhat tilted to the left. The residuals vs. observation order plot graph shows no discernible trend over time. Therefore, it can be concluded from all of these plots that these residuals are independent of one another. This pattern shows that the plots are normally distributed and the errors are unaffected by the prior residuals. According to all of these findings, the ANOVA model offers accurate UTS predictions using the chosen factors, such as LH, PT, and PS.

### 4.2. Microscopic Examination of the Recycled PLA/Wood Specimens

**Figure 7:**
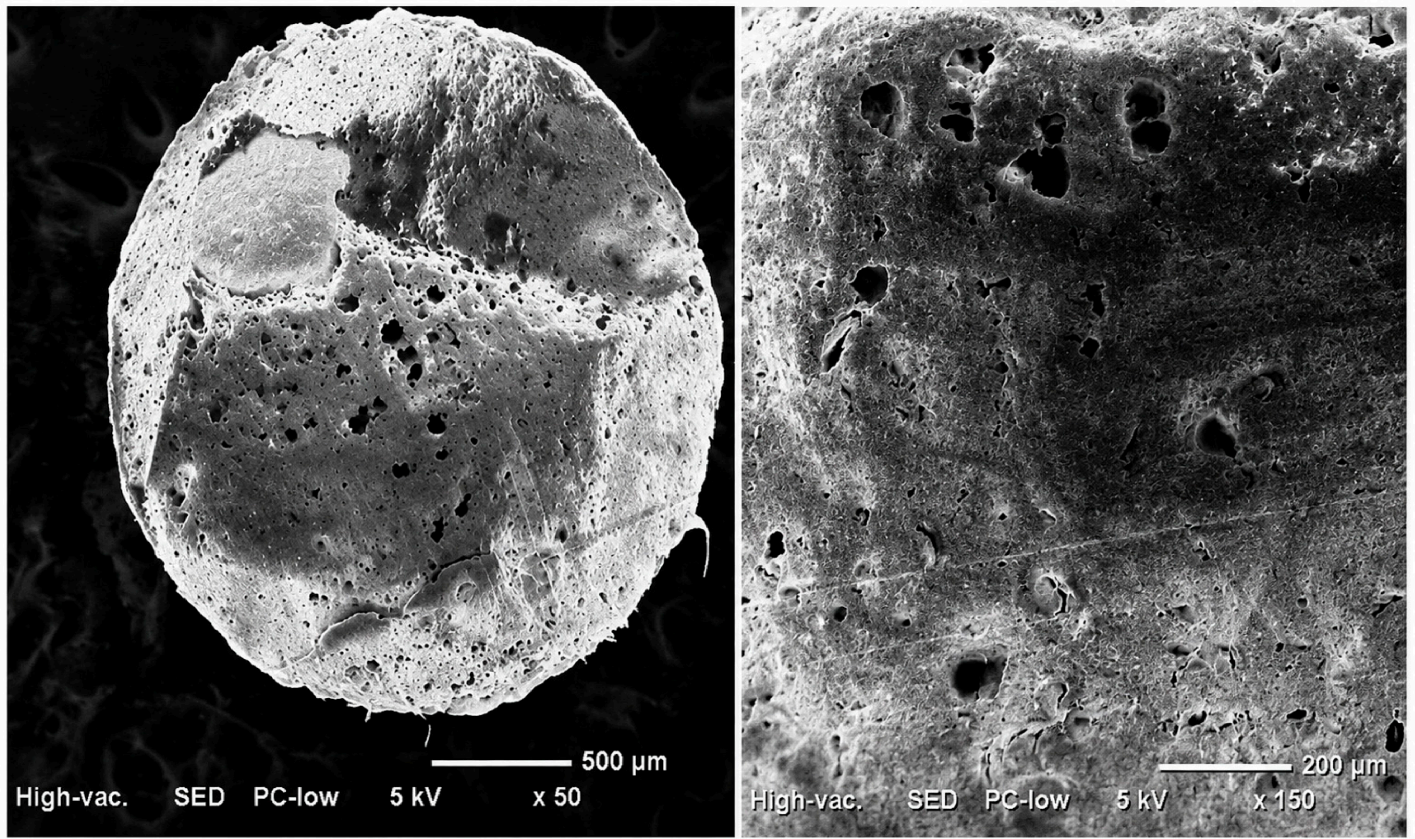
PLA/wood composite filament cross-sectional micrographs.

Tailor-made recycled PLA/wood composite filament cross-sectional micrographs using Scanning Electron Microscopy (SEM) are clearly illustrated in Figure 7. These micrographs reveal the presence of distributed porosity within the filament cross-section. Rounded and irregular voids, ranging from a few micrometers to several tens of micrometers, are observed at both low and high magnifications, reflecting the heterogeneous nature of the PLA/wood composite system. A few voids are observed in the filament cross-section, which is a common feature in 3D-printed filaments produced by melt extrusion. These voids are primarily associated with processing conditions and can be minimized through optimization of the filament extrusion speed, enabling improved melt consolidation.

**Figure 8:**
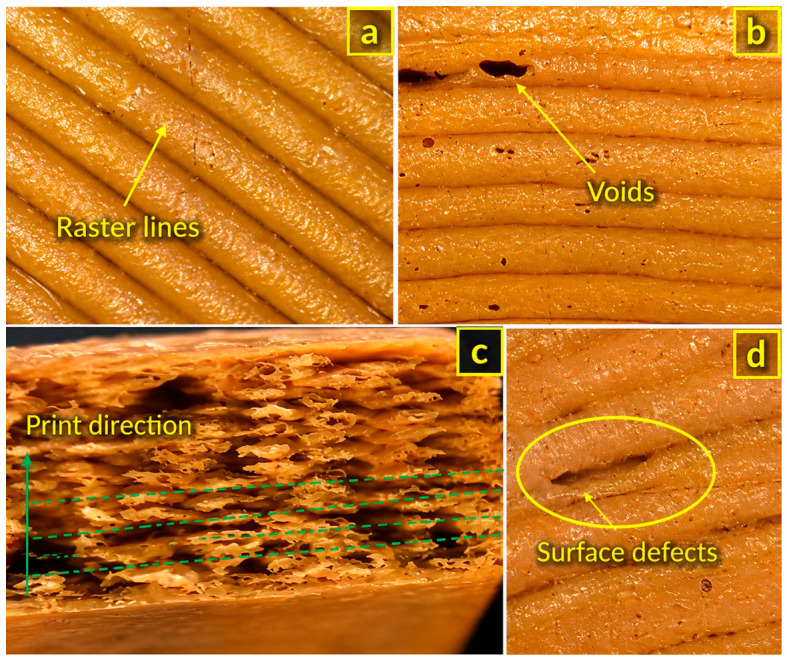
TMM analysis of sustainable recycled PLA/wood composite FFF component (**a**) raster lines; (**b**) voids; (**c**) cross-sectional layered structure and print direction; (**d**) surface defects.

**Figure 9:**
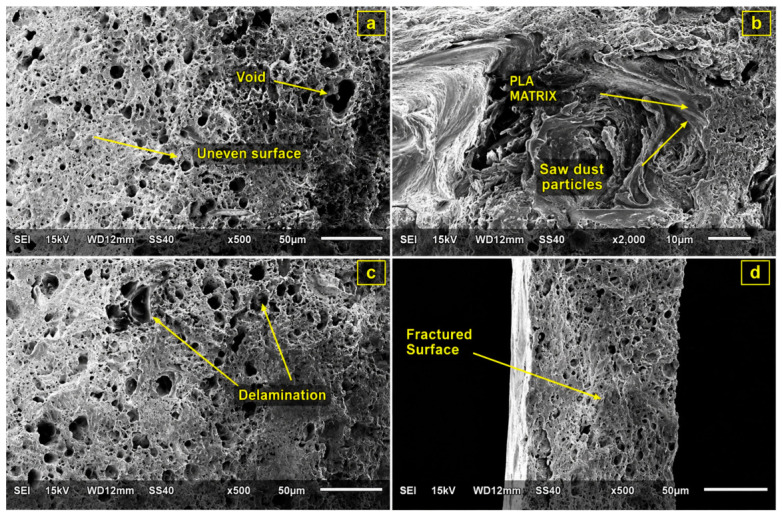
SEM fractography analysis of sustainable recycled PLA/wood composite FFF component (**a**) voids and uneven surface; (**b**) PLA matrix with sawdust particles; (**c**) delamination; (**d**) fractured surface.

Microscopic examinations of 3D-printed recycled PLA/wood components fractography images using SEM and tool makers microscope (TMM) are clearly illustrated in Figure 8 and Figure 9. These analyses provide detailed insights into the internal structure, surface quality, and failure mechanisms of the printed materials. Figure 8a highlights the uniform raster lines on the surface of the FFF tensile specimen, characteristic of the layer-by-layer deposition in FFF. This regularity shows the consistent print pathing; however, surface defects such as voids are also visible in Figure 8b. These voids can result from multiple reasons, including inappropriate process parameter selection, such as low extrusion temperature (ET), insufficient flow rate, or high print speed. Figure 8b distinctly exposes the presence of wood particles embedded in PLA matrix, indicating good filler dispersion in some regions. The contrast in texture between the matrix and reinforcement allows for clear differentiation, suggesting mechanical interlocking between the phases. Figure 8d shows evidence of surface-level delamination, where minor separation between printed layers is observed. This defect is typically associated with weak interlayer adhesion, which is a critical issue in FFF-printed components and significantly affects tensile strength. Figure 8c presents a clear internal view of the fractured specimen using the TMM, with the printing direction indicated by dotted lines. Each line corresponds to a consecutive layer, and the spacing between the lines represents the layer thickness. This image effectively visualizes the layer-wise construction and the anisotropic nature of the composite material. Figure 9a presents the fractographic SEM images of the specimen at fracture region. In this image, the internal voids in between the uniform surface are clearly noticeable. These kinds of voids, generally seen in 3D-printed parts, act as stress concentrators, contributing to crack initiation and premature failure. Figure 9b clearly presents the wood particles in PLA matrix. Figure 9d presents the fractured surface internal micrograph. Figure 9c further confirms the presence of delamination zones within the printed structure. These separations between layers reveal the poor interlayer fusion, often caused by suboptimal printing conditions or improper material settings. This microscopic analysis highlights the critical role of interlayer adhesion, void formation, and filler dispersion in determining the mechanical performance of recycled PLA/wood composites.

### 4.3. Performance Assessment of Various ML Models

The evaluation of the precision and disparities of ML models aimed at estimating the UTS of recycled PLA/wood composites was performed using statistical metrics. This study assesses the effectiveness of ML algorithms utilizing 25 data points, with 80% assigned for training (20 data points) and the remaining 20% for testing (five data points). The analysis employed R^2^, MSE, MAE, MSLE and MAPE assessment metrics for the test dataset of UTS, which comprises five experimental data points. These metrics offer a quantitative assessment of the accuracy with which a ML model corresponds to the actual experimental data.

(12) $$MSE=\frac{1}{n}\sum_{i=1}^{n}{\left(y_{ai}-y_{pi}\right)}^{2}$$

(13) $$R^{2}=1-\frac{\sum_{i=1}^{n}{\left(y_{a}-y_{p}\right)}^{2}}{\sum_{i=1}^{n}{\left(y_{a}-{\bar{y}}_{p}\right)}^{2}}$$

(14) $$\mathrm{MAE}=\sum_{i=1}^{n}\frac{\left|\left(y_{ai}-y_{pi}\right)\right|}{n}$$

(15) $$\mathrm{MSLE}=\frac{1}{n}\sum_{i=1}^{n}{(\log\left(1+y_{ai}\right)-\log\left(1+y_{pi}\right)}^{2})$$

(16) $$\mathrm{MAPE}=\frac{100}{n}\sum_{i=1}^{n}\left|\frac{y_{ai}-y_{pi}}{y_{ai}}\right|$$

where $y_{ai}$—actual outcome and $y_{pi}$—predicted outcome [27,28].

**Figure 10:**
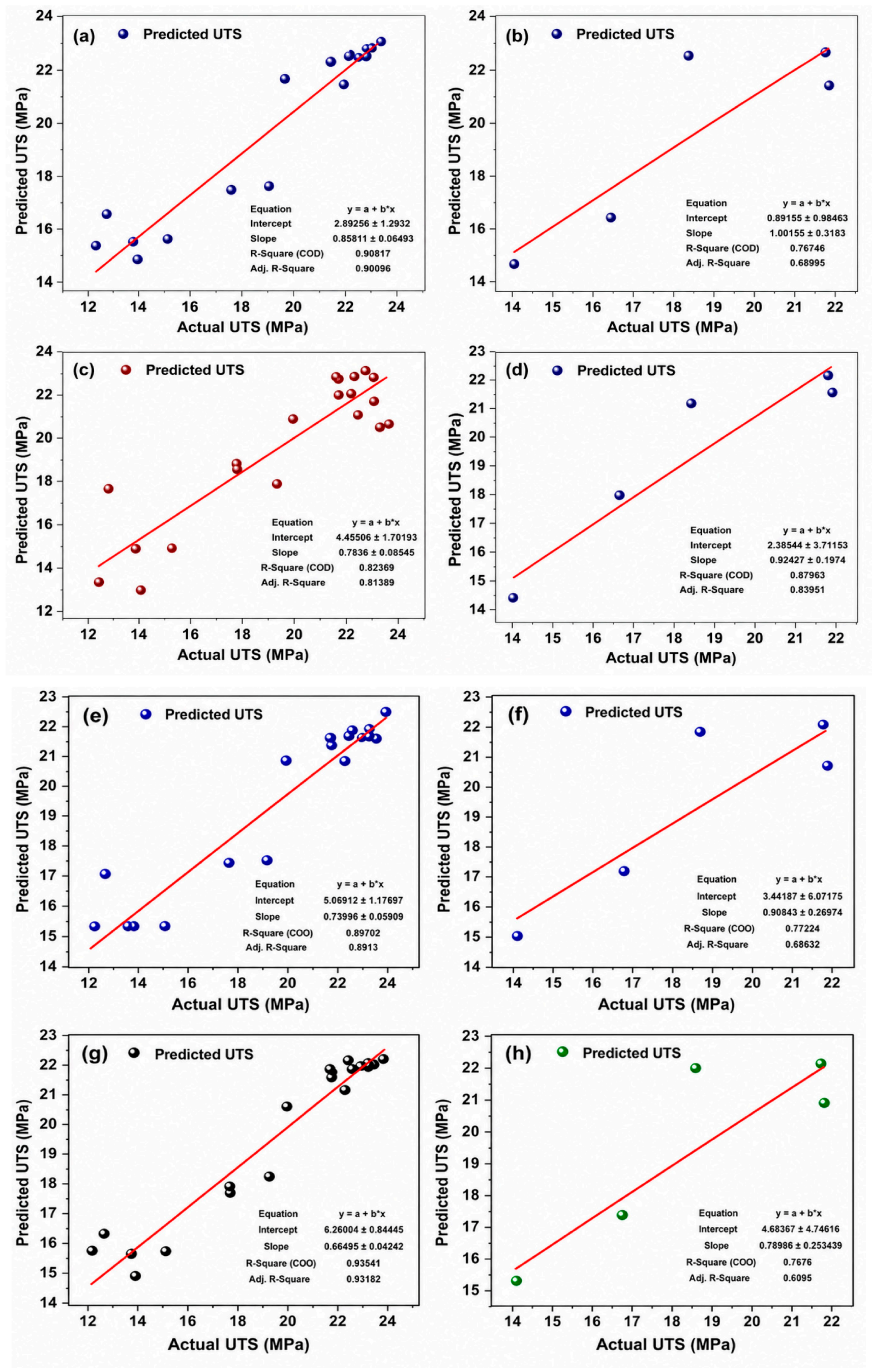

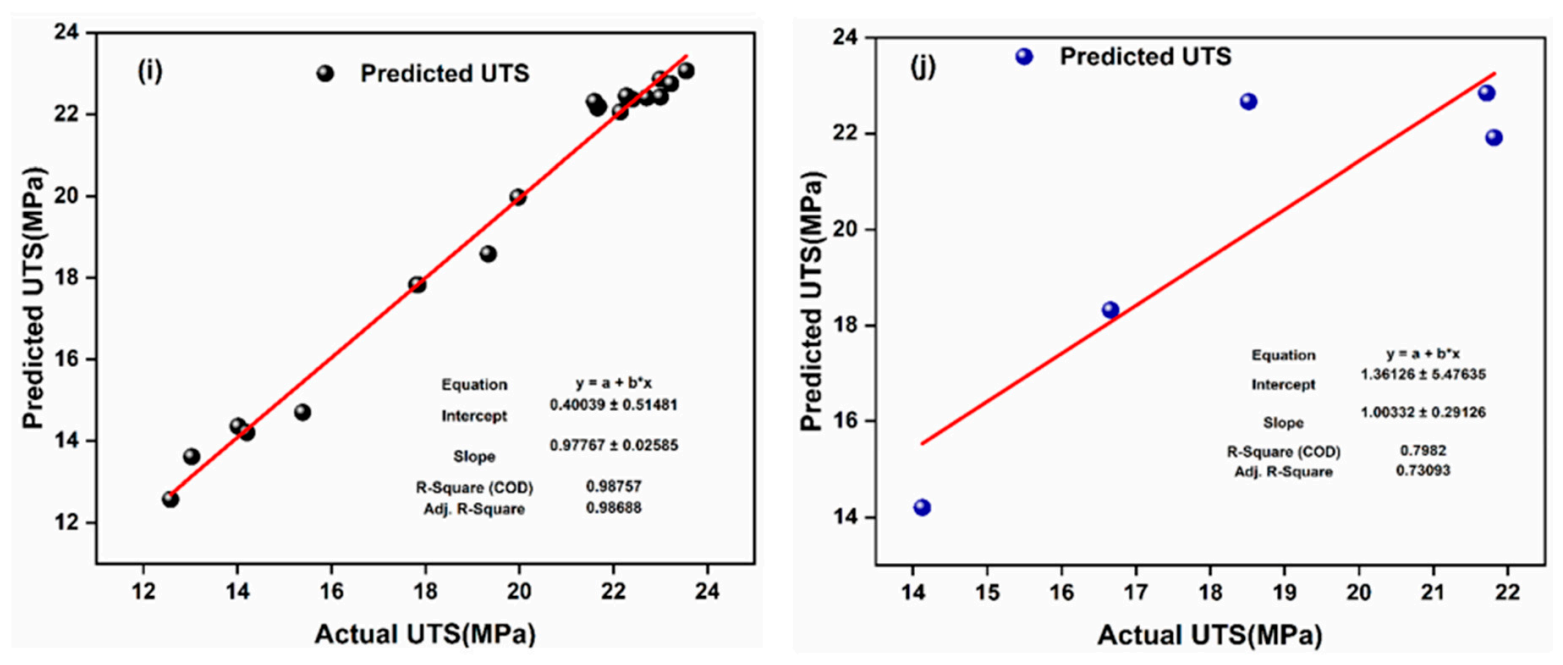
Regression plots of various ML models for training and testing data: (**a**,**b**) RF; (**c**,**d**) SVR; (**e**,**f**) GBR; (**g**,**h**) XGBoost; and (**i**,**j**) Adaboost.

The regression plots shown in Figure 10 demonstrate that, while the ensemble methods (GBR, RF, XGBoost and AdaBoost) demonstrated excellent fitting capability during the training phase, indicated by high R-squared values, they also showed significant drops in their test R-squared values, indicating poor generalization capabilities, and a tendency to be overly complex. Conversely, the SVR model was able to achieve the best R-squared value of any of the models tested, along with nearly unity regression slope and small intercept deviations from unity, and therefore is capable of accurately predicting the scale of the material and having low systematic bias. Further, due to its ability to effectively limit the complexity of the model and handle non-linear behavior well under limited data, the SVR results are seen to tightly cluster around the ideal regression line at virtually all points within the UTS range. Additionally, the relatively small difference between the train and test R-squared values also indicates how robust the SVR model is, and establishes it as the most reliable model for making UTS predictions in this work. The superior testing performance of SVR observed in this study is consistent with prior reports on ML-based prediction of mechanical properties in polymer and composite systems [29]. This study has shown that SVR exhibits strong generalization capability due to its structural risk minimization principle, which effectively controls model complexity and prevents over-fitting, particularly in small- and medium-sized experimental datasets. Unlike ensemble tree-based methods, which tend to favor training accuracy through increased model flexibility, SVR optimizes a margin-based loss function that enhances robustness against noise and data variability. As a result, SVR has been widely reported to outperform or match more complex models in predicting tensile strength, modulus, and fracture properties of polymer composites and bio-based materials when testing data are limited. The highest testing R^2^ and minimal train–test discrepancy obtained in the present work therefore align well with established findings, further confirming the suitability of SVR for reliable UTS prediction in recycled PLA/wood-based composite systems.

### 4.4. Comparison Between Various ML Models

**Figure 11:**
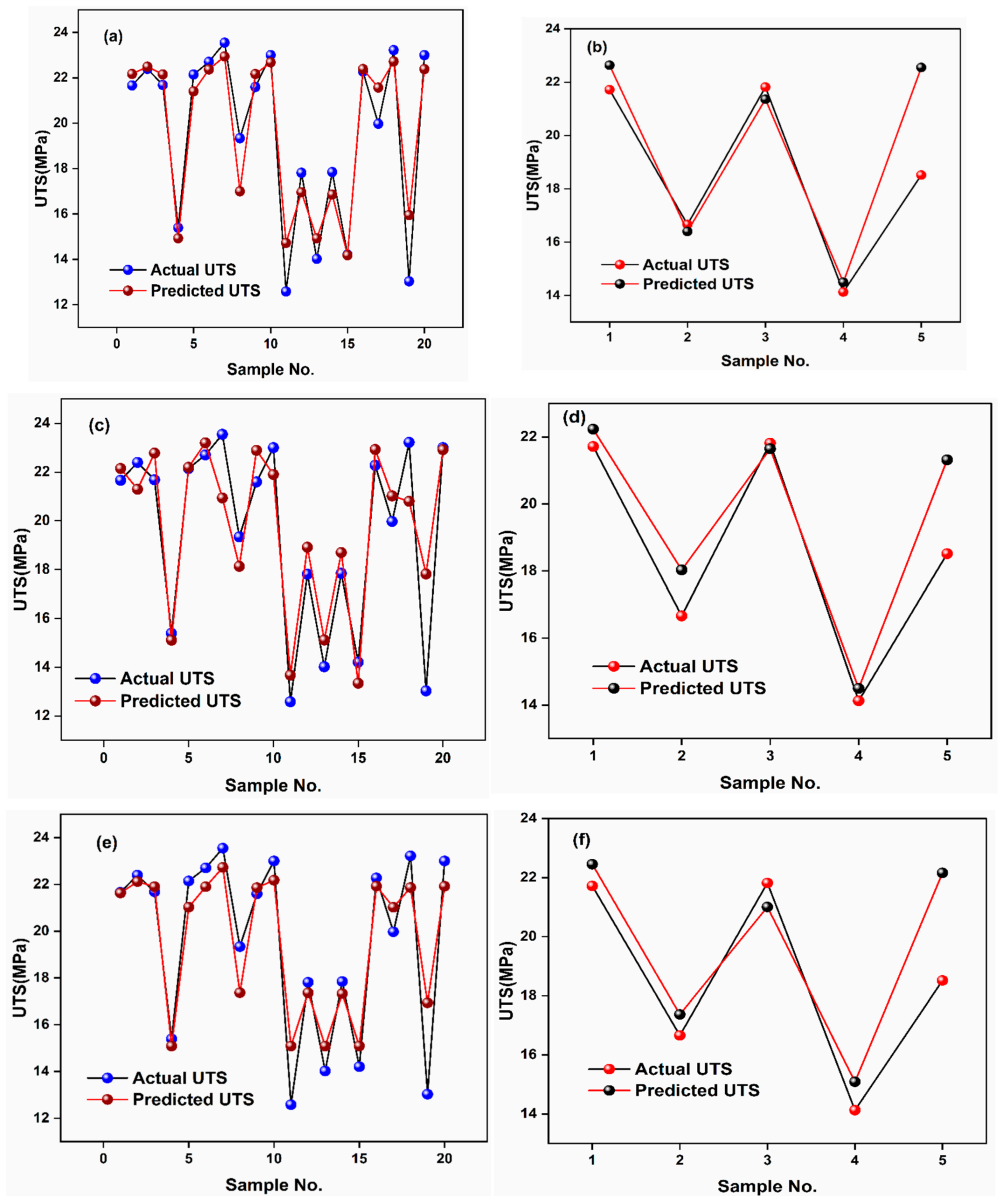

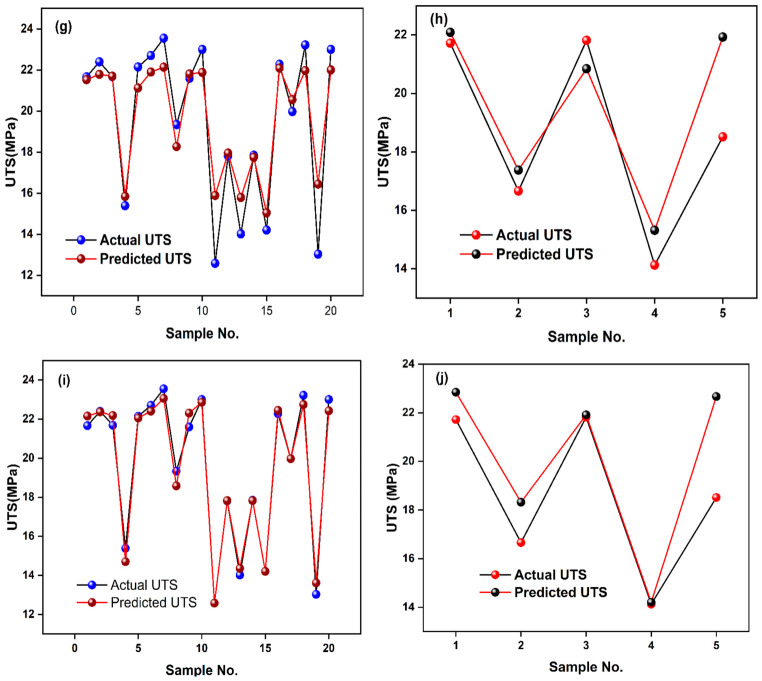
Comparison plots of various ML models for training and testing data: (**a**,**b**) RF; (**c**,**d**) SVR; (**e**,**f**) GBR; (**g**,**h**) XGBoost; and (**i**,**j**) Adaboost.

The relationship between the predictive model’s performance and the experimental results are shown as an ensemble of all of the test and train data in Figure 11. The ensemble models show very low train error and a high degree of variation during testing, which is indicative of a model that has limited ability to be stable or robust. However, the support vector regression (SVR) method displays low prediction bias along with lower variation, and therefore maintains better consistency with the experimentally measured values on an individual sample basis. The SVR method can capture localized variations in UTS while minimizing smoothing; it therefore produces lower point-wise error than other methods for unseen data. The observed behavior is consistent with the known superior stability and reliability of SVR for predicting UTS based on limited experimental data.

**Figure 12:**
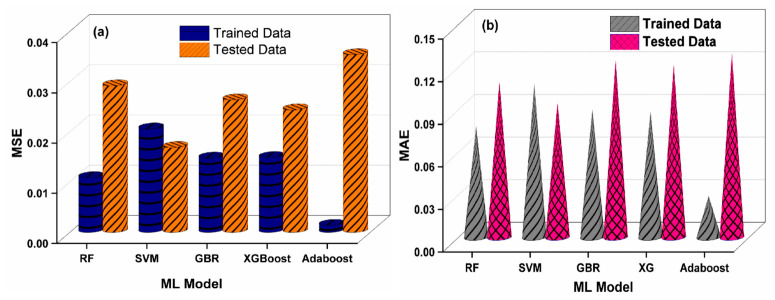

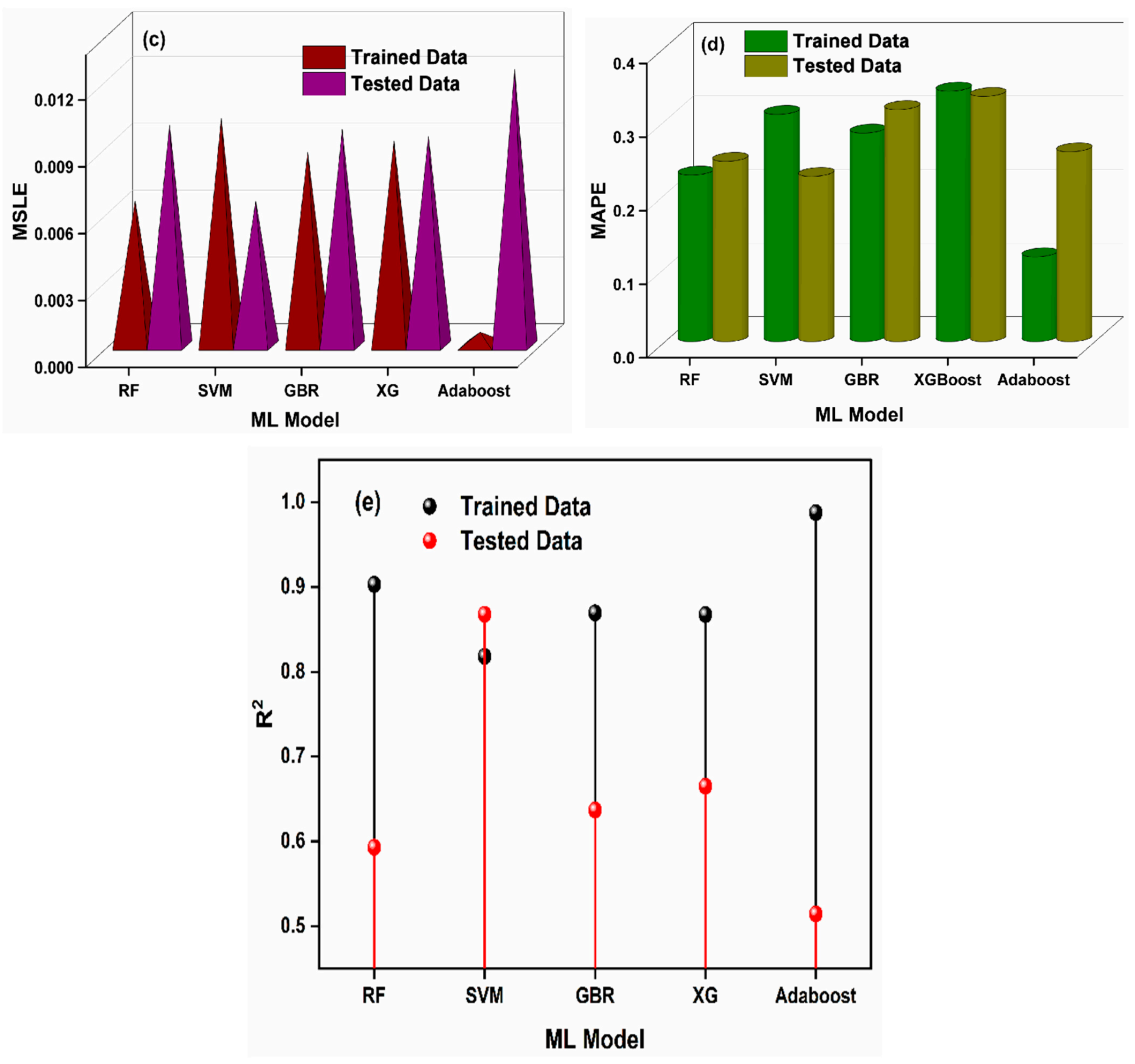
Statistical measures of ML models: (**a**) MSE; (**b**) MAE; (**c**) MSLE; (**d**) MAPE; and (**e**) R^2^.

Figure 12 shows a consistent pronounced train–test performance divergence across all ML models, indicating variance-driven generalization error. The AdaBoost model was found to have the lowest training MSE/MAE/MSLE (training MSE = 0.0014; training MAE = 0.0290); training R^2^ (0.9874). However, the test performance for the AdaBoost model deteriorated significantly (test MSE = 0.0345; test R^2^ = 0.5146) and thus the model appears to be overly fitted and prone to unstable extrapolation. SVM had the best generalization performance as it displayed the highest test R^2^ (0.8679) and the lowest test MSE (test MSE = 0.0169); this demonstrates a superior balance between bias and variance in the models’ performance. Models such as RF, GBR, and XGBoost showed mid-range results in terms of their performance on both training data and test data. While they showed good training results, the test results showed a significant increase in MAE/MAPE; this suggests that these models are sensitive to the amount of local error that is being amplified by the models, and also susceptible to residual heteroskedasticity. In conclusion, the SVM model demonstrated the greatest level of predictive stability, whereas the ensemble boosting models were focused on achieving the “best” possible fit at the cost of generalizability.

In addition to a statistical analysis, the prediction errors were also analyzed based on a practical perspective. As the MAE and MSE indicate how far the calculated UTS are away from the UTS determined through experiments, smaller values indicate better predictive precision. The relatively small test MAE and MSE of the SVR model confirm that the calculated UTS values lie very closely around the related experimental results in terms of the entire examined processing range. Additionally, since the SVR model has the highest test R^2^ compared to all other models, it is confirmed that SVR describes a considerable share of variance within the experimental data, whereas it demonstrates better generalization capabilities than the previously mentioned ensemble-boosting methods. Consequently, the evaluation of R^2^, MAE, and MSE demonstrate that SVR is the most suitable method to predict the UTS of composites produced using an FDM process.

The proposed method is compared to other ML methods used to predict the tensile strength of polymer composite materials made using FDM as shown in Table 6. The majority of prior research has been based on larger datasets than this current work. In addition, they have had access to multiple (4–6) inputs whereas the current study was able to use only three of these inputs. They also were able to utilize a variety of ensemble models which resulted in high accuracy levels of their predictions. On the other hand, the current study was restricted by the fact that there are only 25 samples available for experiments, but it still produced an accurate and reasonable predictive model using only a small number of inputs (three printing parameters: LT, PT, PS) and a simple support vector regression model. The SVR model performed better than all other models tested in terms of its ability to make predictions, both during training and testing (test R^2^ = 0.8679; test MSE = 0.0169), thus showing very good predictive capabilities given the limited amount of data.

## 5. Conclusions

The present study has shown that by combining Design of Experiments (DoE) using the Taguchi approach with ML, an efficient framework to predict the tensile strength of FDM-printed recycled PLA/wood composite parts can be developed. The experimental results show layer height is the most important process parameter affecting ultimate tensile strength while PT has a secondary effect on this property. Conversely, it appears there is a relatively low sensitivity of the tensile properties towards the print speed in the evaluated processing range. Therefore, there seems to be a good degree of freedom to enhance manufacturing productivity with little or no loss of tensile performance.

The relative effectiveness of the five ML algorithms (RF, SVR, GBR, XGBoost, AdaBoost) used to predict ultimate tensile strength of FDM-printed specimens demonstrated considerable differences in both training set accuracy and test set performance. Both the RF and Boosting models provided good fit to the training sets but demonstrated poor generalization from the training to the test sets, demonstrating a potential to “over-fit” the training data. Of all models tested, support vector regression (SVR) provided the best balance between model fitting (training) and model predictive ability (testing) and thus the most reliable predictions of ultimate tensile strength.

Therefore, this research demonstrates that through combining a statistically designed experimental approach with an appropriately selected ML technique, one can develop a method to accurately estimate mechanical properties of materials produced via Additive Manufacturing. Furthermore, this research demonstrates that a combination of statistically designed experiments and ML can greatly reduce the need for extensive experimentation during process development. As such, this research presents a practical method to assist manufacturers in developing optimal processes for producing Additively Manufactured products made from plastic-based polymers.

Future work can focus on detailed microstructural characterization using SEM and other advanced techniques to investigate porosity, reinforcement distribution, interfacial bonding, and fracture mechanisms, thereby providing a deeper understanding of the processing–structure–property relationship.

## Figures and Tables

**Table 1:** Input parameters and their levels.

Input Factor	L1	L2	L3	L4	L5
LH (mm)	0.1	0.15	0.2	0.25	0.3
PT (°C)	190	200	210	220	230
PS (mm/sec)	30	35	40	45	50

**Table 2:** L25 orthogonal experimental layout.

Experiment No.	Layer Height (mm)	Printing Temperature (°C)	Printing Speed (mm/s)	Ultimate Tensile Strength (MPa)
1	0.1	190	30	21.82
2	0.1	200	35	21.69
3	0.1	210	40	22.7
4	0.1	220	45	21.6
5	0.1	230	50	23
6	0.15	190	35	22.15
7	0.15	200	40	23
8	0.15	210	45	22.28
9	0.15	220	50	21.72
10	0.15	230	30	21.66
11	0.2	190	35	19.97
12	0.2	200	40	18.51
13	0.2	210	50	23.55
14	0.2	220	30	22.4
15	0.2	230	35	23.22
16	0.25	190	45	19.34
17	0.25	200	50	16.66
18	0.25	210	30	17.81
19	0.25	220	35	17.85
20	0.25	230	40	13.03
21	0.3	190	50	12.58
22	0.3	200	30	14.02
23	0.3	210	35	15.39
24	0.3	220	40	14.13
25	0.3	230	45	14.2

**Table 3:** Hyperparameters of various ML models.

Model	Parameter	Value
RF	Max_depth	none
	min_samples_leaf	2
	min_samples_split	5
SVR	n_estimators	200
	Regularization parameter (C)	1
	epsilon	1
GBR	Learning rate	0.01
	Max_depth	3
	n_estimators	50
XGBoost	learning_rate	0.01
	max_depth	5
	n_estimators	200
	subsample	0.6
Adaboost	Learning rate	0.01
	loss	exponential
	n_estimators	100

**Table 4:** Taguchi response table.

Response Table for S/N			
Level	LH	PT	PS
1	26.91	25.48	25.69
2	26.91	25.34	25.95
3	26.62	26.06	25.13
4	24.5	25.69	25.55
5	22.94	25.32	25.57
Delta	3.97	0.73	0.81
Rank	1	3	2

**Table 5:** ANOVA table.

Source	DF	Seq.SS	Adj.SS	Adj.MS	F	*p*	Percentage of Contribution
LH	4	63.674	63.674	15.9185	22.24	0	83.91%
PT	4	1.873	1.873	0.4684	0.65	0.635	2.47%
PS	4	1.731	1.731	0.4326	0.6	0.667	2.28%
Residual Error	12	8.587	8.587	0.7156	Residual Error:		11.32%
Total	24	75.865	S = 1.714 R-Sq = 89.0%				

**Table 6:** Comparison of various ML models in the prediction of tensile strength of FDM-based polymer composites.

S.No	Material	Input Features	No. of Features	Sample Size	ML Models	Best Model	Ref.
1	PLA	PT, LH, PS	3	125	RF, LR, SVR, DT, XGBoost, Stacking	XGBoost R^2^ > 0.91	[30]
2	PLA	ID, NT, nozzle diameter (ND), LH, RA and PS	6	329	19 ML regression algorithms	CatBoost: R^2^ = 0.9446; Ensemble: R^2^ = 0.9805	[31]
3	PLA	PS, LH, Wall thickness, NT, ID	5	27	RF, SVR, GPR, XGBoost, MLP, Stacking	ensemble and neural network models R^2^ > 0.99	[31]
4	PLA	LH, PS, ET, ID	4	31	DT, KNN, Gradient Boosting (GB)	KNN Area under curve (AOC) = 0.79, F1 Score = 0.71	[32]
4	Recycled PLA/Wood composite	LH, PT, PS	3	25	RF, SVR, GBR, XGBoost, AdaBoost	SVR: Test R^2^ = 0.8679; Test MSE = 0.0169	[present study]

## Data Availability

The original contributions presented in this study are included in the article. Further inquiries can be directed to the corresponding authors.
